# Lloviu Virus in Europe is an Emerging Disease of Concern

**DOI:** 10.1007/s10393-021-01574-4

**Published:** 2022-02-02

**Authors:** Tamás Görföl, Gábor Endre Tóth, Sándor András Boldogh, Ferenc Jakab, Gábor Kemenesi

**Affiliations:** 1 National Laboratory of Virology, Szentágothai Research Centre, University of Pécs, Ifjúság útja 20, Pécs, 7624 Hungary; 2 Department of Zoology, Hungarian Natural History Museum, Baross utca 13, Budapest, 1088 Hungary; 3 Aggtelek National Park Directorate, Tengerszem oldal 1, Jósvafő, 3758 Hungary; 4 Institute of Biology, Faculty of Sciences, University of Pécs, Ifjúság útja 6, Pécs, 7624 Hungary

Emerging infectious diseases pose an extreme risk for animal populations. Two current devastatingly widespread epizootics, both caused by fungal pathogens, are chytridiomycosis and white-nose syndrome. These infections are responsible for global declines in amphibian and bat populations (Fisher et al. 2012).

Bats are important reservoirs for numerous viruses, including viruses with zoonotic potential (Wang and Anderson 2019). Consequently, an extensive examination of their viral pathogens has been conducted worldwide. Despite this, the only other virus apart from Lyssaviruses which has been reported as a possibly deadly agent for bats is the Lloviu filovirus (LLOV; Negredo et al. 2011). While virus-related bat population declines are considered to be infrequent (O’Shea et al. 2016), the emergence of LLOV as the presumed causative agent of mass mortality events among Schreiber’s bats (*Miniopterus schreibersii*) highlights the risk of viral infections for bats.

The LLOV is a member of the Filoviridae viral family, a family which contains several human pathogens (such as ebolaviruses and Marburg virus) that can cause disease with high mortality rates for humans and non-human primates (Emanuel et al. 2018). Several studies suggest the reservoir role of bats for Filoviruses (e.g. Olival and Hayman 2014) and an unexpected phylogeographic diversity of bat-borne Filoviruses were revealed during the past few years (e.g. Yang et al. 2017).

In the early 2000s, the LLOV presumably caused mass mortalities in Schreiber’s bat population in Spain, Portugal, and France (Negredo et al. 2011). After more than a decade, LLOV re-emerged in 2016 in Hungary along with two additional possibly connected mass mortality events from 2013 to 2016 (Kemenesi et al. 2018). In addition, we recently confirmed the presence of the virus in dead animals from January of 2019 and living bats in autumn 2019 at one previously affected roost in Hungary, which presented evidence for the continuous circulation of LLOV there. In Spain, LLOV seroprevalence studies showed the circulation of the virus in healthy bats captured in 2015 in caves where LLOV was originally identified (Ramirez de Arellano et al. 2019). During the recorded die-off episodes in Hungary, more than 600 animals—five per cent of the Hungarian population—of Schreiber’s bats perished. Recently, with the successful isolation of LLOV on a bat cell line and proving its ability to infect monkey and human cells, the zoonotic potential of the virus was confirmed (Kemenesi et al. 2021).

**Figure 1:**
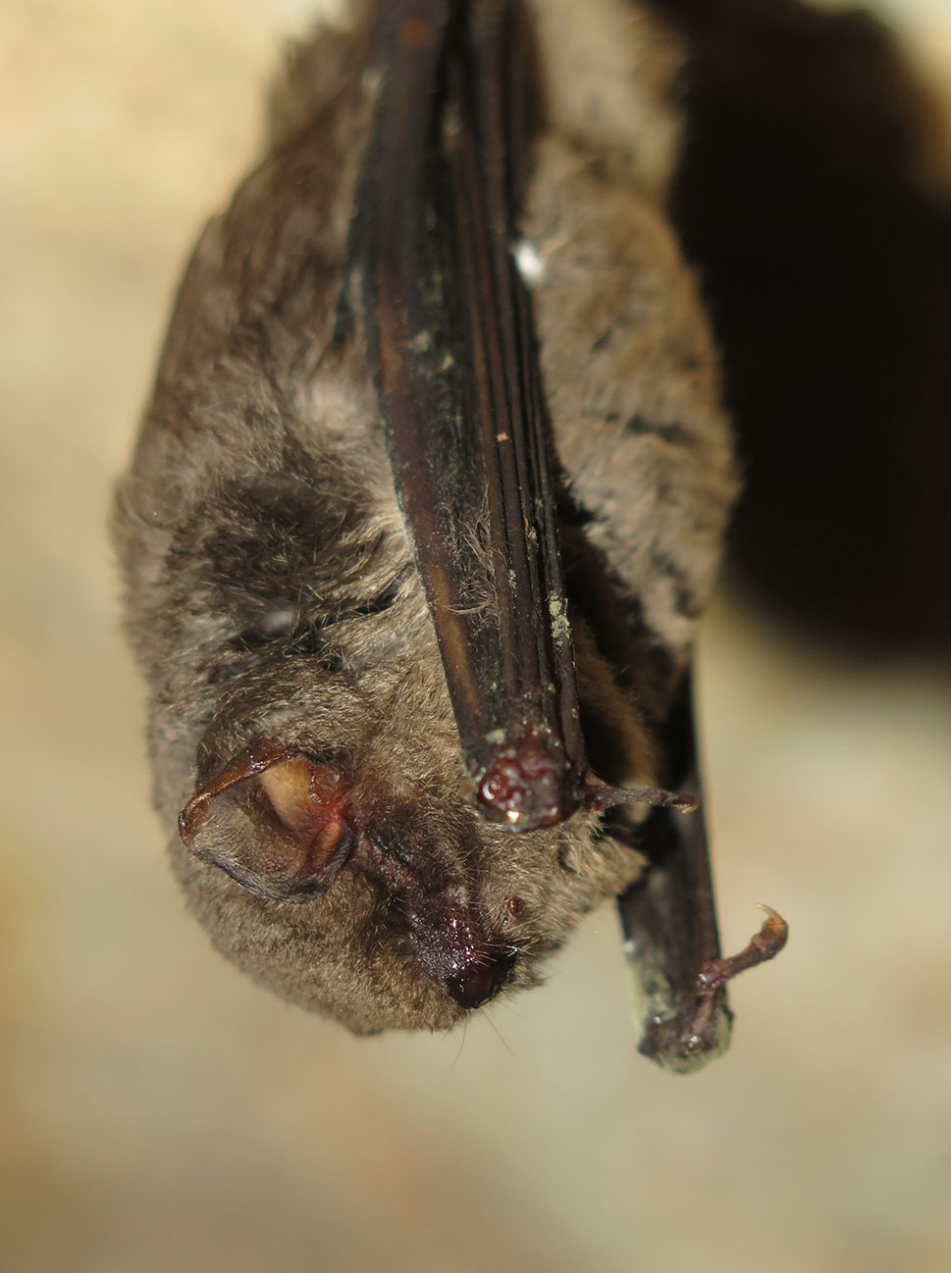
A dead *Miniopterus schreibersii* bat at the affected mine.

Several unique symptoms were observed such as haemorrhagic signs around the mouth and nose in some individuals (Fig. 1); bats hibernating in a relaxed pose; and hanging from only a single leg. In all the suspected cases, a generally poor physical condition (e.g. depleted fat reserves) was observed. Since the first emergence in 2013, several dozen animals remained in an affected mine during winter periods, while the majority (3000–7000 animals) of the summer colony continued to migrate as normal to a traditional and yet unknown winter roost. The lack of information about this issue is concerning, particularly given the species’ declining population trend, and the fact that it forms large aggregates in the few available roosting sites remaining. The exact impact of the virus on the population dynamics of the species is still unclear and may become a prominent direction of future studies.

More broadly, our findings underscore the potential of viral pathogens to severely affect bat populations, causing mass mortalities, similarly to *Pseudogymnoascus destructans* causing white-nose syndrome epizootics. RNA viruses—like Filoviruses—are among pathogens that are the most likely to jump between hosts, and therefore posing a direct risk of human infections (Woolhouse et al. 2005).

A multi-lateral approach must be applied to reduce negative effects on bat populations, and to prevent human infections:

- Molecular and serological study of bats, their ectoparasites and the environment may unfold the source(s) of LLOV and reveal transmission pathways.
- Population genetic study of known hosts (e.g. *Miniopterus schreibersii*) is needed to get a clearer picture about the possible directions of the geographic spreading of the virus.
- While there is a knowledge gap on the potential reservoirs of LLOV, prevention of possible human infection is crucial. The overall prohibition of visiting Schreiber’s bat roosts (beside scientific purposes) must be prescribed.
- Wearing personal protective equipment when visiting roosts and handling bats is important. As LLOV is a possible zoonotic pathogen, in case of hazardous situations (e.g. finding of dead Schreiber’s bats), using appropriate personal protective equipment according to the possible presence of a biosafety level 4 agent is critically important.
- When epizootic event occurs, immediate closing of the site and its neighbouring roosts from humans as well as disinfection of all caving and sampling material is the most important first-step mitigation measure.
- Regular monitoring of bat roosts must be accompanied at least for Schreiber’s bat colonies, to be able to immediately react when die-offs occur.
- As a relative of the Ebola virus, communication about LLOV—the only Filovirus naturally occurring in Europe—may lead to biased public perception about bats, and may seriously affect future conservation efforts of habitats and colonies, hence maintaining public awareness must be a priority.

To resolve the unknown details of the threats posed by the re-emergence and circulation of LLOV, we encourage a multinational collaboration of researchers from different disciplines (virologists, conservation biologists, and bat researchers), and increased research into this issue. LLOV is not the first and we believe that not the last viral agent that may pose a significant threat to bats and humans; hence, the abovementioned procedures may be applicable to the mitigation of future bat-borne viral epizootics or spillover events as well.
